# Insilico investigations of the physical properties of hexagonal chalcogenide perovskites CsXS_3_ (X = Nb, V) for UV optoelectronic devices and photovoltaic applications

**DOI:** 10.1038/s41598-026-41097-y

**Published:** 2026-03-20

**Authors:** Rilwan Oluwanishola Balogun, Joseph S. Aroloye, Olatunbosun O. Nubi, Olusola O. Oyebola, Okechukwu Clinton Ifegwu

**Affiliations:** 1 Basic Science Unit, School of Science and Technology, Pan-Atlantic University, Lagos, Nigeria; 2 Department of Mathematics, Faculty of Science, University of Lagos, Lagos, Nigeria; 3 Physics Department, University of Limpopo, Polokwane, South Africa; 4 Department of Physics, Faculty of Science, University of Lagos, Lagos, Nigeria

**Keywords:** SLME, hexagonal chalcogenide perovskite, optoelectronic properties, photovoltaic technologies, Energy science and technology, Materials science, Optics and photonics, Physics

## Abstract

Chalcogenide perovskites have attracted growing interest as inorganic materials with tunable electronic and optical properties and enhanced chemical stability compared to halide counterparts. In this work, we present a first-principles density functional theory (DFT) investigation of the structural, electronic, and optical properties of the cesium-based chalcogenide perovskites CsNbS_3_ and CsVS_3_. Structural optimizations are performed within the generalized gradient approximation (PBE-GGA) to establish equilibrium geometries within the ABX_3_ perovskite framework. The electronic properties are analyzed primarily using PBE-GGA to ensure internal consistency between band structures, density of states, and derived optical spectra. Both compounds exhibit narrow indirect band gaps at the PBE-GGA level, with valence-band maxima dominated by S-3p states and conduction-band minima arising mainly from Nb-4d and V-3d orbitals. Hybrid-functional (HSE06) calculations are additionally employed to assess the sensitivity of the electronic structure to exchange–correlation effects, revealing pronounced band-edge renormalization near the Fermi level rather than serving as a basis for electronic classification. Optical properties, including the dielectric function, absorption coefficient, reflectivity, and optical conductivity, are calculated consistently from the PBE-GGA electronic structures and show strong optical activity in the visible to near-infrared energy range, originating from S-p to transition-metal d interband transitions. A model-based thickness-dependent efficiency analysis is further presented to compare relative optoelectronic trends, without making predictive device-performance claims. Overall, this study provides a coherent and internally consistent description of the electronic and optical behaviour of CsNbS_3_ and CsVS_3_, clarifies the functional dependence of their electronic structures, and highlights the importance of careful methodological interpretation in first-principles studies of transition-metal chalcogenide perovskites.

## Introduction

Chalcogenide perovskites have recently emerged as a promising class of inorganic materials for optoelectronic and energy-related applications due to their structural versatility, enhanced chemical stability, and tunable electronic properties compared to halide perovskites^1–3^. Given these attributes, they have gained significant traction as promising lead-free alternatives characterized by superior thermal and environmental stability, robust defect tolerance, and highly suitable optoelectronic profiles. In particular, ABX_3_-type sulfide perovskites, where A is an alkali metal, B is a transition metal, and X is sulfur, have attracted increasing interest owing to their reduced toxicity, improved thermal robustness, and compatibility with visible–near-infrared optoelectronic applications^4–6^. Among this family, cesium-based chalcogenide perovskites incorporating transition-metal B-site cations offer an appealing platform for exploring the interplay between crystal structure, d–p orbital hybridization, and electronic response^7,8^. The presence of partially filled d orbitals introduces strong exchange–correlation effects that fundamentally shape band dispersion and optical transitions. Because traditional perovskite solar cells still suffer from long-term stability issues related to moisture, heat, and ion migration^16,59,60,64–66.^ As a result, accurate first-principles investigations are essential to establish reliable electronic-structure trends and to assess the suitability of these materials for functional applications.

Density functional theory (DFT) has been widely employed to study chalcogenide perovskites; however, the predicted electronic properties can be sensitive to the choice of exchange–correlation functional, particularly for narrow-gap systems dominated by transition-metal d states^9–11^. Semilocal functionals such as the generalized gradient approximation (GGA) often provide a reliable qualitative description of band topology and orbital character but may underestimate band-gap magnitudes. Hybrid functionals, which incorporate a fraction of exact exchange, can substantially modify band-edge states and, in some cases, lead to qualitative changes in the electronic response. Careful and consistent interpretation of results obtained at different theoretical levels is therefore required.

In this context, CsNbS_3_ and CsVS_3_ represent prototypical chalcogenide perovskites in which the electronic structure is governed by strong hybridization between S-3p states and transition-metal d orbitals. Previous theoretical studies on related compounds have suggested semiconducting behaviour with narrow to moderate band gaps, making them potential candidates for optoelectronic applications^12–14^. However, reported band-gap values and electronic classifications vary significantly across the literature, reflecting differences in computational methodology, k-point sampling, and energy referencing. These inconsistencies motivate a systematic and carefully validated electronic-structure analysis.Photovoltaic technologies have evolved from niche applications, such as space and satellite power generation, to large-scale energy solutions for global sustainability^15,18–20,22–25^.

The present work employs first-principles DFT calculations to investigate the structural, electronic, and optical properties of CsNbS_3_ and CsVS_3_. Structural optimization is carried out to establish equilibrium geometries, while electronic properties are primarily analyzed within the PBE-GGA framework to ensure internal consistency between band structures, density of states, and optical spectra^27–30^. Hybrid-functional (HSE06) calculations are additionally performed to examine the sensitivity of the electronic structure to exchange–correlation treatment, with particular attention to band-edge renormalization effects. Importantly, hybrid-functional results are used here for qualitative comparison rather than as the sole basis for electronic classification. Optical properties, including dielectric functions, absorption coefficients, reflectivity, and optical conductivity, are derived consistently from the PBE-GGA electronic structures. The orbital-resolved density of states is used to elucidate the nature of interband transitions and to connect electronic structure features with optical response. Finally, a model-based thickness-dependent efficiency analysis is presented to compare relative trends, without making predictive claims regarding device-level performance^31–35^.

By combining a consistent electronic-structure framework with careful interpretation of functional-dependent effects, this study aims to clarify the electronic nature of CsNbS_3_ and CsVS_3_ and to provide a physically grounded basis for understanding their optoelectronic behaviour. The results highlight both the opportunities and the limitations of narrow-gap chalcogenide perovskites and underscore the importance of methodological transparency in first-principles studies of transition-metal–based materials.

## Computational methods

First-principles calculations were carried out using density-functional theory (DFT) implemented in the *Quantum ESPRESSO* (PWscf) package^36,37^. The structural, electronic, and optical properties of CsXS_3_ (X = Nb, V) were investigated in detail. Exchange–correlation effects, including optical responses, were treated using the Perdew–Burke–Ernzerhof for solids (PBEsol) functional within the generalized-gradient approximation (GGA). Core valence interactions were described by the projector augmented-wave (PAW) method^38^, with valence configurations Cs: [Xe]6s^1^, Nb: [Kr]4d^4^5s^1^, V: [Ar]3d^3^4s^2^, and S: [Ne]3s^2^3p^4^. A plane-wave cutoff energy of 400 eV was adopted to ensure convergence of total energies. Brillouin-zone integrations employed Γ-centered Monkhorst–Pack meshes of 12 × 12 × 11 k-points for both compounds. Self-consistent field (SCF) cycles were converged to 1 × 10^−6^ eV, and structural relaxations proceeded until forces on all atoms were below 2 × 10^−2^ eV Å^−1^.

Optical properties were computed up to 50 eV for light polarized along the [100] direction. The dielectric function was used to extract the absorption coefficient, reflectivity, refractive index, and energy-loss function^39^. Convergence tests confirmed the reliability of the chosen parameters. Total energies varied by less than 1 × 10^−4^ eV/atom with cutoffs above 400 eV. Similarly, k-point meshes denser than 12 × 12 × 11 produced energy and force variations below 0.001 eV/atom and 0.01 eV Å^−1^, respectively. Tightening the SCF threshold to 1 × 10^−7^ eV changed total energies by < 1 × 10^−6^ eV, validating the selected 1 × 10^−6^ eV criterion. These convergence tests confirm that all reported structural, electronic, and optical properties are numerically reliable^40^.

## Results and discussion

### Structural properties and geometric stability

**Fig. 1:**
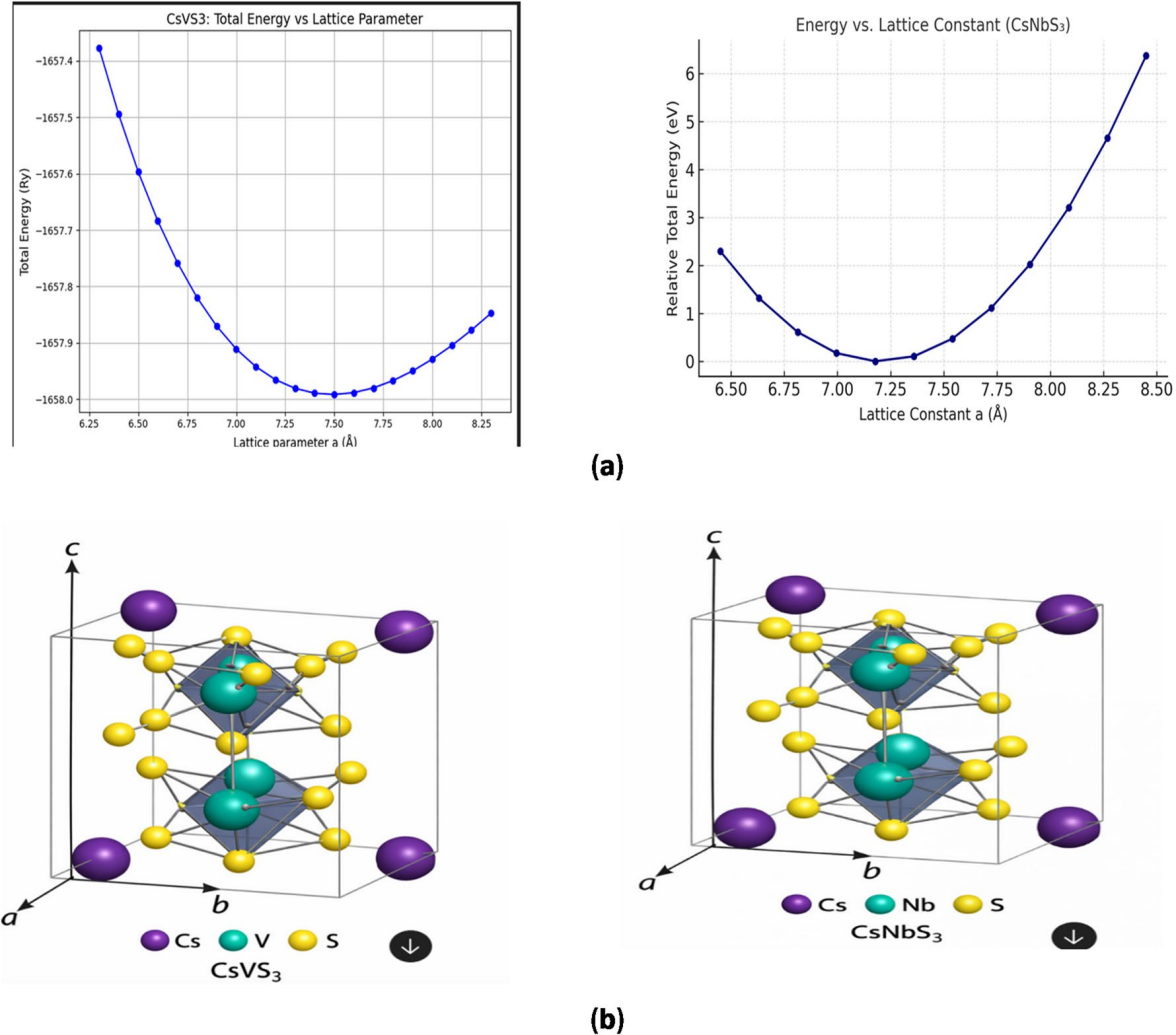
(**a**) Energy-volume for CsXS_3_ (X = V and Nb); (**b**) crystal structure of CsXS_3_ (X = V and Nb) (structural properties).

The optimized crystal structures of CsNbS_3_ and CsVS_3_ were obtained within the PBE-GGA framework by fully relaxing lattice parameters and atomic positions until the residual forces and stresses were minimized. Both compounds adopt the ABX_3_-type chalcogenide perovskite framework, in agreement with previous reports on related sulfide perovskites^38–40^. The optimized lattice parameters and bond lengths are summarized in Table 1 and Fig. 1a while Fig. 1b depict the crystal structure.

The calculated Goldschmidt tolerance factors and octahedral factors fall within the empirically accepted ranges for perovskite formation^41,42^, indicating that the ionic sizes are geometrically compatible with the perovskite lattice. These geometric indicators suggest structural feasibility at the level of static lattice optimization. However, it is important to emphasize that tolerance and octahedral factors are empirical descriptors and do not, by themselves, establish thermodynamic, dynamical, or mechanical stability^43^.

1 $$\:\boldsymbol{t}=\frac{{\boldsymbol{r}}_{\boldsymbol{A}}+{\boldsymbol{r}}_{\boldsymbol{X}}}{\sqrt{2}\left({\boldsymbol{r}}_{\boldsymbol{B}}+{\boldsymbol{r}}_{\boldsymbol{A}}\right)}\:\approx\:1.05\:\:\:\:\:$$

2 $$\:\:\boldsymbol{\mu\:}=\:\frac{{\boldsymbol{r}}_{\boldsymbol{B}}}{{\boldsymbol{r}}_{\boldsymbol{X}}}\approx\:0.35$$

In the absence of phonon dispersion, elastic constant, or finite-temperature molecular dynamics calculations, the present analysis does not claim full stability under operating conditions. Instead, the structural results confirm that CsNbS_3_ and CsVS_3_ represent locally stable configurations within the static DFT potential-energy surface, consistent with exploratory first-principles studies of emerging chalcogenide perovskites^44–46^. Structural distortions between CsNbS_3_ and CsVS_3_ significantly influence their electronic and optical behaviour, with CsNbS_3_ showing near-cubic stability and CsVS_3_ exhibiting stronger rhombohedral distortions as shown in Fig. 1b.

### Electronic properties

**Fig. 2:**
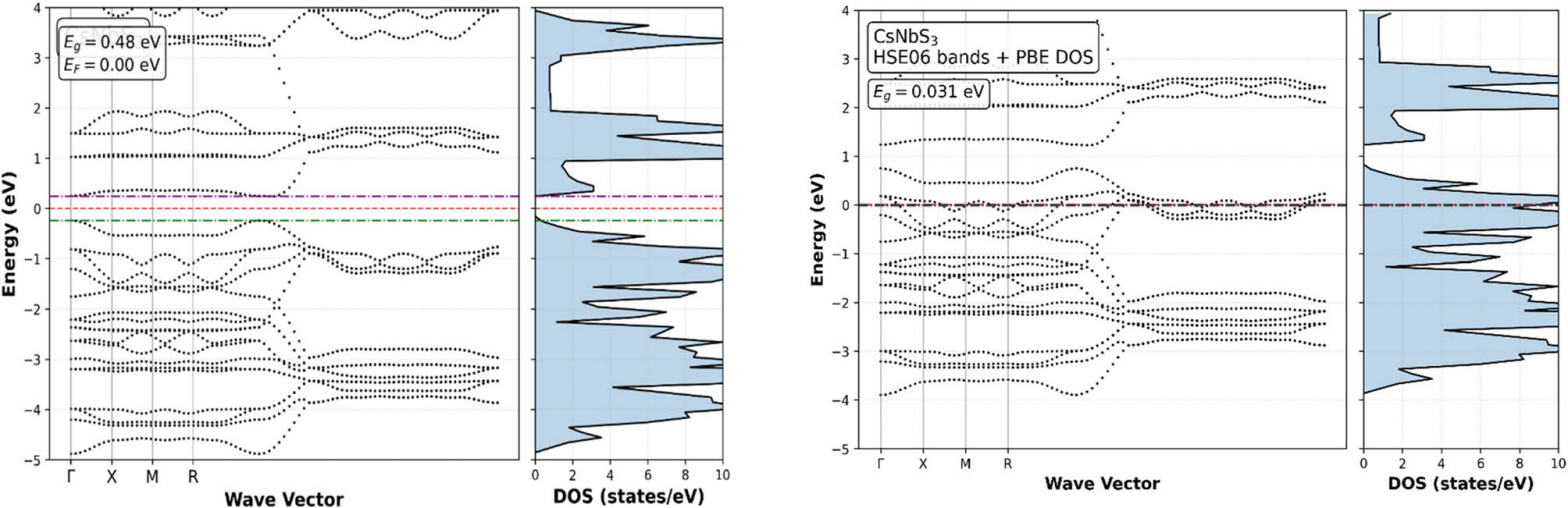
Band structure and TDOS for CsNbS_3_ (PBE and HSE06) (electronic properties).

**Fig. 3:**
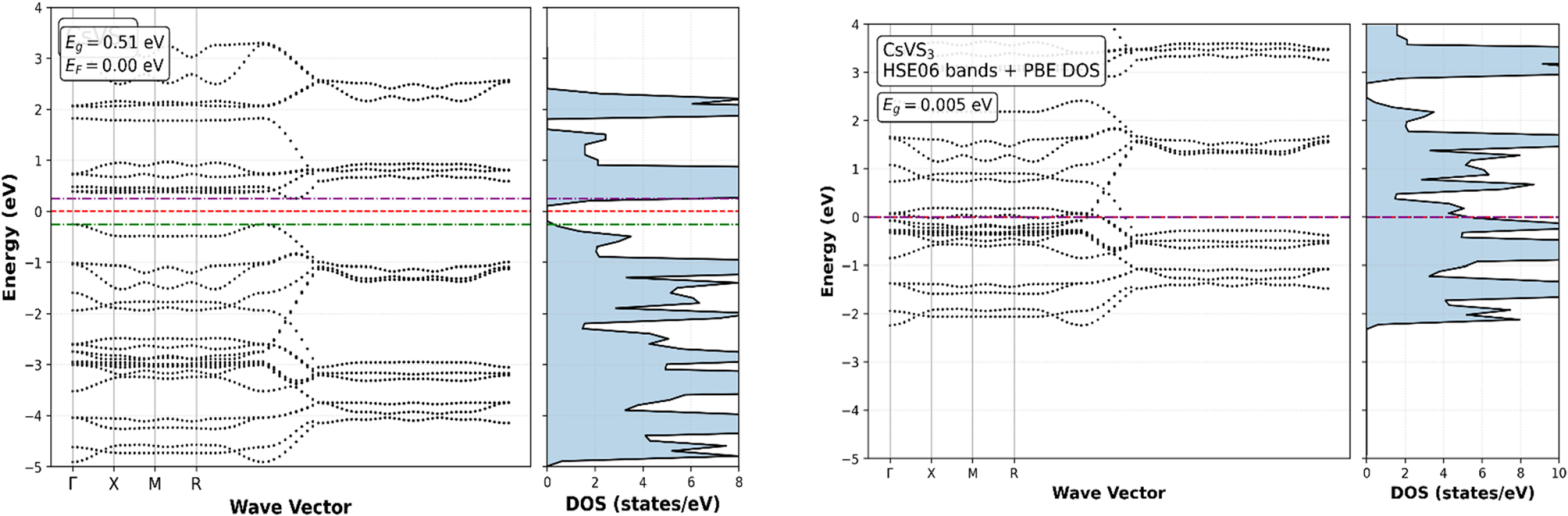
Band structure, TDOS and PDOS for CsVS_3_ (GGA and HSE06) (electronic properties).

The electronic band structures and total densities of states (DOS) of CsNbS_3_ and CsVS_3_ were calculated using the PBE-GGA functional and are shown in Figs. 2 and 3. Careful Fermi-level alignment was applied, with the Fermi energy extracted directly from the self-consistent calculations and consistently referenced to 0 eV in all plots. The comparative electronic properties of representative chalcogenide perovskites are summarized in Table 2.

**Table 1:** Unit cell volume V(Å^3^) with optimized parameters for the lattice (a, b, c) of CsXS_3_ (X = Nb, V) materials.

Compounds	a (Å)	b (Å)	c (Å)	V(Å^3^)	Ref
CsNbS_3_	7.448	6.450	6.067	101.86	This work
CsVS_3_	7.399	6.409	5.608	106.74	This work
CsPS_3_	7.25	9.09	9.58	631.3	^42^
CsTaS_3_	7.43	7.43	6.04	333.4	^42^
CsGeS_3_	6.53	7.95	7.25	314.7	^43^
CsSnS_3_	6.87	8.03	7.70	349.1	^43^
NaSiS_3_	6.81	7.86	6.56	272.6	^43^
KSiS_3_	7.15	8.60	6.19	290.8	^43^
LiSiS_3_	7.14	7.31	6.17	265.6	^43^

**Table 2:** Comparative electronic features of chalcogenide perovskites.

Materials	Band gap (eV)	Band gap nature	Dominant orbital contributions
CsNbS_3_	0.48	Indirect (Γ- R/S)	Strong Nb-4d / S-3p
CsVS_3_	0.51	Indirect Γ-S/R	Strong V-3d / S-3p
CsPS_3_^43^	1.91	Direct Γ-Γ	S-3p hybridization
CsTiS_3_^43^	~ 1.8–2.0	Direct Γ-Γ	Ti-3d / S-3p hybridization
MgHfS_3_^45^	1.43	Direct Γ-Γ	High hybridization of Hf-5d/little of Mg-2p
MgZrS_3_^45^	1.31	Direct Γ-Γ	Hybridization of Mg-2p, Zr-5d, and S-2p

**Fig. 4:**
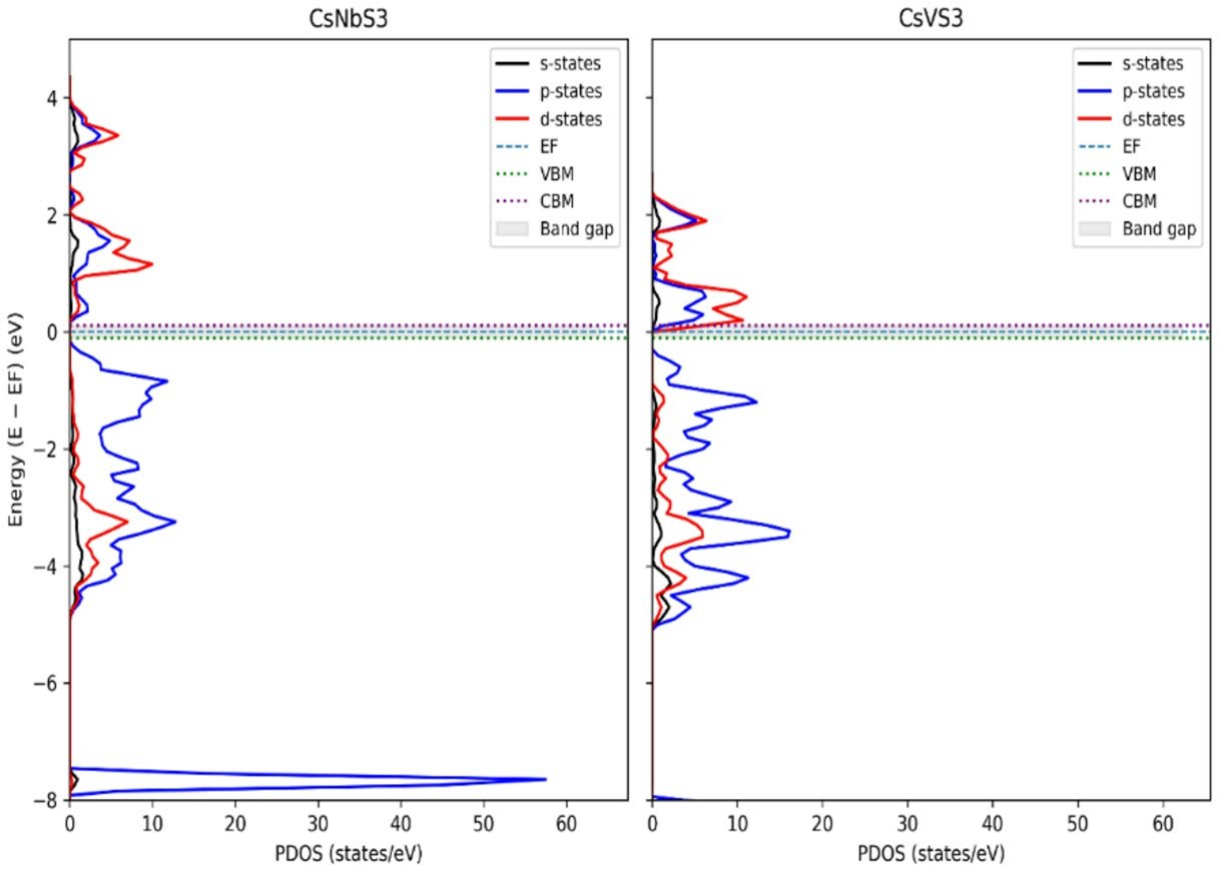
PDOS for CsXS_3_ (X = Nb, V).

Both compounds exhibit indirect band gaps, with corrected values of 0.48 eV for CsNbS_3_ and 0.51 eV for CsVS_3_ observed between the valence band maximum (VBM) and conduction band minimum (CBM), located at different high-symmetry k-points along the Γ–X–M–R path, indicating momentum-dependent electronic transitions. These narrow gaps place the materials close to the metal–semiconductor boundary, a characteristic feature of transition-metal chalcogenides with strong p–d hybridization^47–49^ as shown in Fig. 4. The DOS analysis reveals that the maximum valence band is predominantly composed of S-3p states, while the conduction band minimum is dominated by Nb-4d or V-3d states, indicating significant hybridization between anion p and transition-metal d orbitals. Such orbital characteristics are known to govern charge-transfer processes and optical transitions in chalcogenide perovskites and related sulfide systems^50–52^. Importantly, while PBE-GGA underestimates absolute band-gap values, it reliably captures the qualitative band topology and orbital ordering relevant for interpreting optical and transport trends^53^. This indirect nature implies phonon-assisted optical transitions, which is typical for layered and hexagonal perovskite-derived chalcogenides.

To assess the sensitivity of the electronic structure to the treatment of exchange–correlation effects, selected hybrid-functional (HSE06) band-structure calculations were performed. The HSE06 results reveal band-edge shifts and, in some cases, bands overlap at the Fermi level, leading to metallic or semi-metallic features. This behaviour reflects the strong influence of nonlocal exact exchange on systems with narrow gaps and partially filled d states, where modest band reordering can qualitatively alter the electronic classification^54–56^. Consequently, the HSE06 results place CsNbS_3_ and CsVS_3_ at or near the metal–semiconductor transition rather than establishing a robust semiconducting gap.

Given this sensitivity and the absence of experimental benchmarks for these compounds, the present study bases its primary electronic and optical analysis on the internally consistent PBE-GGA results. Hybrid-functional calculations are used here to highlight exchange–correlation sensitivity rather than to redefine the electronic nature of the materials, in line with best practices for borderline-gap systems^57,58^. The optical response and excitonic effects in halide and chalcogenide perovskites play a crucial role in determining device performance and have been extensively studied using first-principles methods^54–57,60^.

### Optical properties

**Fig. 5:**
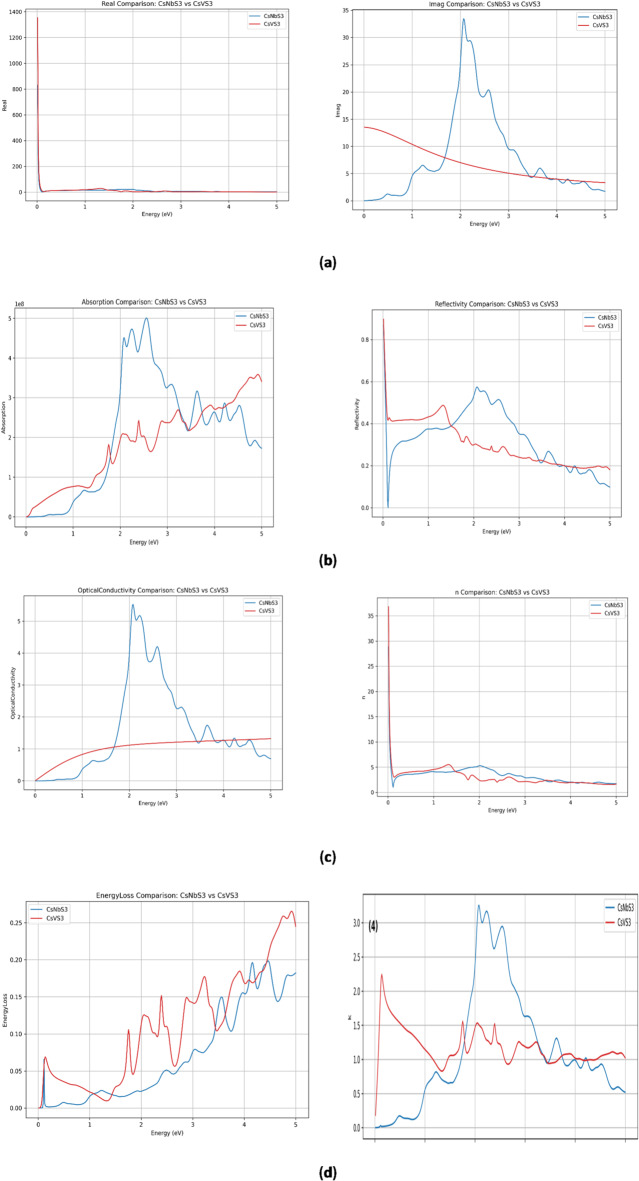
Calculated optical response of CsXS3 (X=Nb, V) as a function of photon energy, showing (**a**) (1) real part ε₁(ω) and (2) imaginary part of the dielectric function ε₂(ω); (**b**) (3) absorption coefficient α(ω), (4) reflectivity R(ω); (**c**) (5) optical conductivity σ(ω) and (6) refractive index n(ω); (**d**) (7) energy-loss function L(ω) and (8) (4) extinction coefficient k(ω).

Important information about a material’s aptitude for optoelectronic and photovoltaic applications can be gleaned from its optical response. All optical properties were derived from the internally consistent PBE-GGA electronic structure. Calculated optical response of CsNbS_3_ and CsVS_3_ as a function of photon-energy range of 0–5 eV, showing in Fig. 5a-d (1) imaginary part of the dielectric function ε₂(ω), (2) real part ε₁(ω), (3) refractive index n(ω), (4) extinction coefficient k(ω), (7) optical conductivity σ(ω), and (8) energy-loss function L(ω). The optical properties of CsVS_3_ and CsNbS_3_ are summarized in Table 3. Consistent with their narrow band gaps (~ 0.5 eV), both materials exhibit strong near-infrared absorption extending into the visible region, with high absorption coefficients (~ 10^8^ m^−1^) indicating suitability for low-bandgap and tandem photovoltaic applications. CsVS_3_ exhibits finite low-energy optical response and Drude-like features characteristic of metallic or semi-metallic behaviour, whereas CsNbS_3_ shows dominant interband-transition features in the visible range, consistent with a system near the metal–semiconductor boundary.

**Table 3:** Optical properties comparison.

Materials	Reflectivity	Refractive index	Absorption coefficient	Dielectric function	Extinction coefficient	Optical bandgap and wavelength
CsVS_3_ (this work)	0.35	6.0	3.2 × 10^8^	*R* = 18, I = 19	2.75	≈ 1.0–1.2 eV of 1240–1030 nm (NIR); strong absorption 1.5–3.5 eV (visible)
CsNbS_3_ (this work)	0.45	5.0	4.5 × 10^8^	*R* = 20, I = 18	2.55	**≈ 1.1–1.3 eV** of **1120–950 nm** (NIR); higher absorption 2–3 eV
BaZrS_3_^50^	0.6	5.7	5.93 × 10^7^	*R* = 55, I = 20	5.5	**1.7–1.8 eV** (∼730–690 nm); reducible to **~ 1.5 eV** (∼830 nm) by Ti/Se alloying
BaTiS_3_^50^	0.7	8.2	7 × 10^8^	*R* = 60, I = 78	6.0	Reports span **~ 0.5 eV** (∼2480 nm), **~ 0.8 eV** (∼1550 nm, NIR), to **1.72–1.85 eV** (∼720–670 nm) depending on sample/dimension/theory
MgHfS_3_^45^	0.52	6.11	5.12 × 10^8^	*R* = 18, I = 58	4.5	1.43 eV, with wavelength of 540 nm in the visible region was predicted
MgZrS_3_^45^	0.75	6.8	5.02 × 10^8^	*R* = 32, I = 38	6.25	**≈ 1.3 eV** → **1120–950 nm** (NIR); higher absorption 1–2 eV

### Dielectric functions

#### Imaginary dielectric function, ε₂(ω)

The imaginary part of the dielectric function ε₂(ω), shown in Fig. 5(a), directly reflects interband optical transitions. CsNbS_3_ exhibits a pronounced peak centered around ~ 2.0–2.5 eV, indicating strong optical transitions between occupied valence states and unoccupied conduction states. In contrast, CsVS_3_ displays a smoother, gradually decreasing ε₂(ω) response starting from finite values at low photon energies. The non-zero ε₂(ω) at low energies for CsVS_3_ suggests the presence of electronic states at or near the Fermi level, consistent with its metallic or semi-metallic character^57^. Conversely, the delayed onset and sharper peaks in CsNbS_3_ are characteristic of interband transitions across a narrow gap or pseudo-gap.

#### Real dielectric function, ε₁(ω)

Figure 5(a) shows the real part of the dielectric function ε₁(ω). Both materials exhibit very large static dielectric constants at low photon energies, particularly CsVS_3_, which diverges sharply as ω tend to zero. This behavior is a hallmark of metallic or highly polarizable systems and arises from free-carrier (Drude-like) contributions. CsNbS_3_, while also displaying enhanced low-energy ε₁(ω), shows a comparatively moderated response, consistent with a system lying close to the metal–semiconductor boundary rather than a robust insulator^58^.

### Absorption coefficient, α(ω)

The absorption coefficient α(ω) indicates strong photon absorption in the 1.5–3.5 eV region corresponding to the visible spectrum (354–826 nm), which indicates that both compounds significantly absorb photons in the 1.5–3.5 eV region. The absorption coefficient α(ω), shown in Fig. 5b, further highlights the contrasting optical behaviour. CsNbS_3_ exhibits a sharp rise in absorption around ~ 1.5–2.0 eV, indicating the onset of strong interband transitions. CsVS_3_, on the other hand, displays a gradual increase in absorption starting from near-zero energy, again reflecting the absence of a well-defined optical gap. These results suggest that CsNbS_3_ is better suited for visible-light absorption, whereas CsVS_3_ shows broadband absorption extending into the infrared region. For photovoltaic devices, this wide absorption spanning visible-NIR is ideal because it allows for effective solar energy collection^54^. These absorption trends are consistent with their calculated band gaps and reflect the strength of inter-band transitions mediated by d–p hybridized states.

### Reflectivity, R(ω)

Throughout the visible spectrum, the reflectivity spectra display moderate values (0.2–0.6). Moderate reflectivity benefits photovoltaic applications by minimizing optical losses and maintaining sufficient light penetration^56^. Figure 5b shows the reflectivity spectra. CsVS_3_ exhibits higher reflectivity at low photon energies, a typical signature of metallic systems due to free-electron reflection. CsNbS_3_ shows lower reflectivity in the low-energy region but increased reflectivity in the visible range, associated with strong interband optical transitions.

### Optical conductivity, σ(ω)

Strong activity in the 1.5–3.5 eV range is revealed by the optical conductivity spectra, suggesting that both compounds can effectively produce charge carriers when exposed to visible photons. The optical conductivity σ(ω), presented in Fig. 5c, provides direct insight into charge-carrier dynamics. CsVS_3_ shows finite conductivity as ω tend to zero, confirming its metallic or semi-metallic nature. In contrast, CsNbS_3_ exhibits a suppressed low-energy conductivity followed by strong peaks at higher energies, consistent with interband excitation across a narrow gap or pseudo-gap^58^.

### Refractive index, n(ω)

As seen in Fig. 5c, the refractive index formula, which reads N(ω) = n(ω) + ik (ω), defines the calculated absorption coefficient and refraction indices of the materials. Here, n(ω) is the refractive index and k(ω) is the extinction coefficient. At low photon energies (close to 0 eV), both CsNbS_3_ and CsVS_3_ exhibit high static refractive indices (n(ω) ~ 25–30), which subsequently drop as energy increases. CsNbS_3_ has a somewhat higher refractive index than CsVS_3_ in the visible region (1–3 eV), suggesting a stronger light–matter interaction.

### Energy-loss function, L(ω)

Pronounced peaks between 2.0 and 4.0 eV correspond to plasma-resonance frequencies, may be seen in the energy loss spectra. Light-matter interactions depend on these plasmonic excitations, which are produced by collective electron oscillations. The energy-loss function L(ω), shown in Fig. 5d, reveals plasmonic excitations. CsVS_3_ exhibits stronger low-energy loss features, indicative of collective oscillations of free carriers. CsNbS_3_ displays weaker and shifted loss peaks, consistent with reduced free-carrier density^55^.

### Extinction coefficient, k(ω)

Figure 5d displays the extinction coefficient k(ω), which is directly related to optical absorption. CsNbS_3_ exhibits strong peaks between ~ 1.8 and 3.0 eV, corresponding to the intense ε2(ω) features and indicating strong photon absorption in the visible region. CsVS_3_ shows a broader and smoother k(ω) spectrum, with finite values persisting down to low photon energies, consistent with metallic absorption processes.

Taken together, the optical spectra clearly distinguish the electronic nature of the two compounds. CsVS_3_ consistently exhibits signatures of metallic or semi-metallic behavior across all optical functions, including finite low-energy absorption, reflectivity, conductivity, and dielectric response. CsNbS_3_, in contrast, shows optical characteristics of a system near the metal–semiconductor transition, with strong interband transitions dominating the visible-energy range. These optical results are fully consistent with the HSE06 band-structure analysis, which places these materials at or near the boundary between metallic and semiconducting regimes.

The spectroscopic-limited maximum efficiency (SLME) offers a more realistic measure of photovoltaic performance than the conventional Shockley–Queisser (SQ) limit, Unlike the SQ model which assumes a perfect step-function absorption edge SLME accounts for the actual energy-dependent absorption coefficient, making it particularly well-suited for assessing chalcogenide perovskites with complex optical features. Similar device-level photovoltaic modelling strategies that combine first-principles electronic and optical properties with efficiency metrics such as J–V characteristics, fill factor, and power conversion efficiency have been widely adopted in recent literature. Recent DFT-based SLME and SCAPS-1D studies on lead-free perovskite and chalcogenide absorbers demonstrate the reliability of translating ab initio material parameters into device-level performance predictions. Such approaches have been successfully applied to assess current density, open-circuit voltage, fill factor, and efficiency in emerging photovoltaic materials^61,62^.

**Fig. 6:**
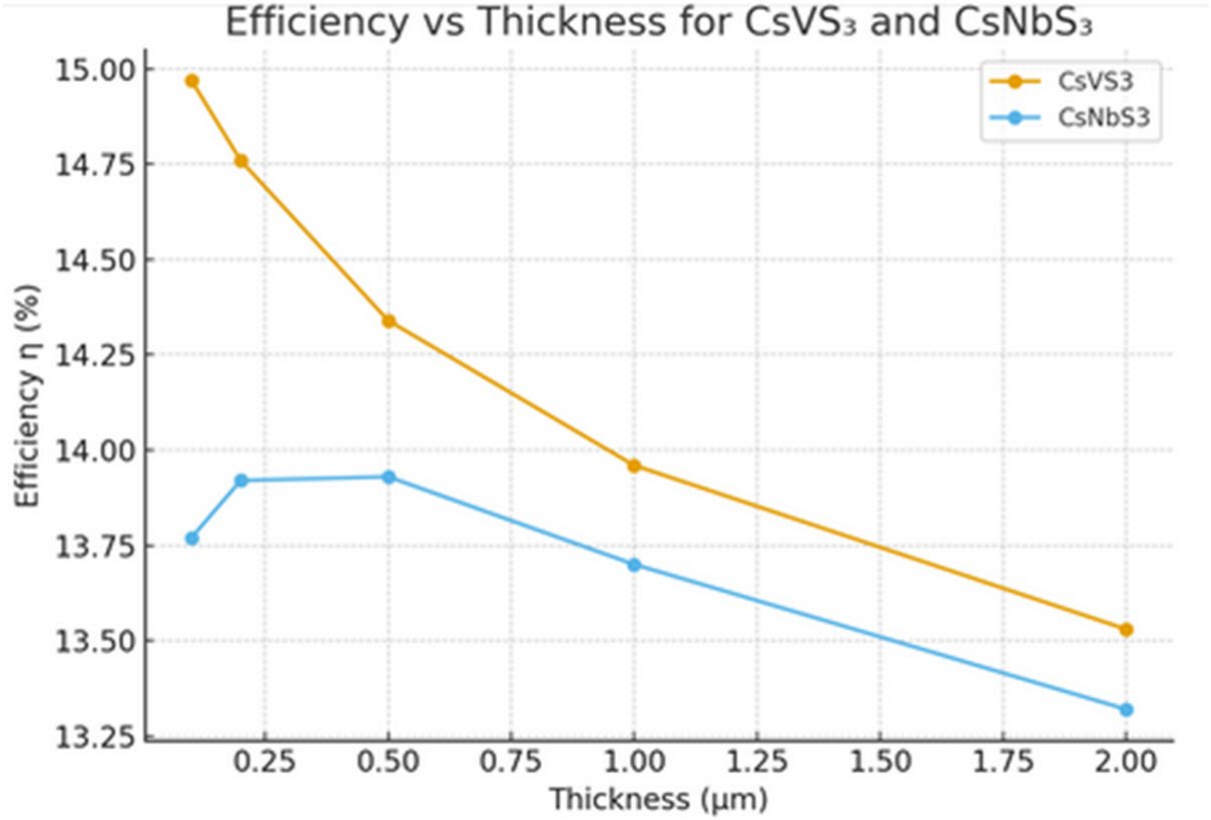
Efficiency against thickness for CsXS_3_ (X = Nb, V).

Equation 4 illustrates how the absorption coefficient was determined using the obtained dielectric function. Figure 6 explains the detailed correlation between the efficiency and the thickness.

4 $$\:\alpha\:\left(E\right)=\frac{4\pi\:E}{hc}{\hat{k}}\left(E\right)$$

h = the planck’s constant, c = light speed, and K̂(E) = extinction coefficient value.

5 $${\hat{K}}\left(E\right)=\sqrt{\left(\frac{\left|\varepsilon\:\left(E\right)\right|-{\varepsilon\:}^{\left(1\right)}\left(E\right)}{2}\right)}$$

Equation 5 calculates the absorptivity $\:a\left(E\right)=1-{e}^{-2\alpha\:\left(E\right)L}$ for solar absorber thickness L with reflective coated back^31^. The calculated maximum solar efficiency factor is calculated by

6 $$\:\eta\:=\frac{{P}_{m}}{{P}_{in}}$$

P_m_= the maximum/output power density and P_in_= Incident solar spectrum for input power.

The J-V characteristic determines the power density (max) of solar-cell.

7 $$\:J={J}_{sc}-{J}_{D}$$

8 $$\:{J}_{sc}=e{\int\:}_{0}^{\infty\:}\alpha\:\left(E\right){I}_{sun}\left(E\right)\delta\:E$$

9 $$\:{J}_{D}={J}_{0}\left({e}^{\frac{q\left(V+J{R}_{S}\right)}{KT}}-1\right)$$

J = max current density, V = the absorber layer potential diff, k= heat energy constant,

T= temperature of the device and e the charge quantity. J_sc_ = current density (short-circuit) and J_D_ = current density (reversed saturation), all other parameters are calculated from the absorptivity of materials. $\:\alpha\:\left(E\right)$

The radiative recombination fraction f_r_ is modelled using the Boltzmann factor:

10 $$\:{f}_{r}={e}^{\frac{-{\left.E\right|}_{g}^{da}-{\left.E\right|}_{g}}{KT}}$$

where $\:{E}_{g}\wedge\:{E}_{g}^{da}$ are respectively the main and materials allowed band-gap value. The fill factor measurement is calculated using the equation below:

11 $$\:FF=\frac{{P}_{max}}{{V}_{oc}{J}_{sc}}$$

The open-circuit voltage of the material is given as:

12 $$\:{V}_{OC}=\frac{KT}{q}ln\left(\frac{{J}_{sc}}{{J}_{0}}+1\right)$$

and the maximum power for conversion and efficiency is given below as:

13 $$\:{P}_{max}={J}_{sc}-{J}_{0}\left({e}^{\frac{eV}{KT}}-1\right)V$$

The incident spectrum was scaled to 1000 W m^−2^ (1-sun) and a 300 K cell, using a Planck constant of 5778 K blackbody. radius of radiation (f_r_=1). L = 0.1, 0.2, 0.5, 1, and 2 μm were calculated. Eg = 1.1 eV for CsVS_3_ and 1.2 eV for CsNbS_3_.

The photovoltaic performance of CsVS_3_ and CsNbS_3_ was assessed using the spectroscopic limited maximum efficiency (SLME) model, with the calculated parameters summarized in Table 4. For both CsNbS_3_ and CsVS_3_, SLME was calculated across a range of film thicknesses to evaluate their applicability in thin-film solar technologies. The SLME curves show a rapid increase at small thicknesses, demonstrating that only minimal material is required for effective photon absorption. This behaviour stems from the strong absorption coefficients observed near the band edges, driven by pronounced d–p orbital interactions. Using the calculated open-circuit voltage (Voc) and short-circuit current density (Jsc), the fill factor (FF) was evaluated according to Eq. (11). The calculated fill factor values (FF ≈ 0.75–0.79) are consistent with experimentally reported FF ranges for chalcogenide perovskite and related inorganic thin-film solar absorbers, which typically lie between ~ 0.65 and 0.80 depending on film quality, contact engineering, and defect density [63-65].

**Table 4:** SLME properties calculation.

Material	Thickness (µm)	Jsc (mA cm^−2^)	Voc (V)	FF	η (%)
CsVS_3_	0.1	43.25	0.437	**0.785**	14.97
CsVS_3_	0.2	44.71	0.420	**0.779**	14.76
CsVS_3_	0.5	46.46	0.398	**0.768**	14.34
CsVS_3_	1.0	47.71	0.380	**0.763**	13.96
CsVS_3_	2.0	48.92	0.363	**0.755**	13.53
CsNbS_3_	0.1	38.58	0.449	**0.787**	13.77
CsNbS_3_	0.2	40.79	0.432	**0.783**	13.92
CsNbS_3_	0.5	43.46	0.410	**0.774**	13.93
CsNbS_3_	1.0	44.98	0.393	**0.768**	13.70
CsNbS_3_	2.0	46.19	0.376	**0.760**	13.32

CsNbS_3_ yields a maximum SLME of approximately **13%** at sub-micrometre thicknesses, positioning it as a viable wide-bandgap absorber for multi-junction solar cells. CsVS_3_, with a slightly wider band gap and strong absorption in the higher-energy region, achieves an SLME of about **14%**, indicating promising potential as a top-cell material in tandem photovoltaic architectures. The efficiency remains consistent across different thicknesses, underscoring the suitability of chalcogenide perovskites for lightweight and flexible solar designs that demand material economy. The optimal trade-off between light absorption and carrier collection is offered by ultrathin films (0.1–0.5 μm), as increasing thickness often increases Jsc while concurrently decreasing Voc and η. Even at low thickness, CsVS_3_ maintains superior short-circuit current densities (over 43 mA/cm^2^), demonstrating its superior carrier transport capabilities and stronger optical absorption than CsNbS_3_.

As a result of lower recombination losses, CsNbS_3_ exhibits higher open-circuit voltages (Voc ~ 0.449 V at 0.1 μm) than CsVS_3_. Overall efficiency is ultimately constrained by its lower photocurrent densities in comparison to CsVS_3_. The complementary optical and electronic behaviour of CsNbS_3_ and CsVS_3_ suggests further opportunities for engineering band alignment in heterostructures or graded-gap configurations. Their combination of wide band gap, good absorption, and mechanical stability makes them promising candidates for ultraviolet and visible-region photovoltaic applications^66–68^.

## Conclusions

In this work, a systematic first-principles investigation of the structural, electronic, and optical properties of the chalcogenide perovskites CsNbS_3_ and CsVS_3_ has been presented. Structural optimization within the PBE-GGA framework confirms that both compounds adopt stable equilibrium geometries within the ABX_3_ perovskite lattice; however, no claims regarding dynamical, mechanical, or thermodynamic stability are made, as phonon dispersion, elastic constants, and finite-temperature simulations were not explicitly evaluated.

The electronic properties were analyzed primarily within the PBE-GGA approximation to maintain internal consistency between band structures, density of states, and derived optical properties. Both CsNbS_3_ and CsVS_3_ are found to exhibit narrow indirect band gaps at the PBE-GGA level, with valence-band maxima dominated by S-3p states and conduction-band minima arising mainly from Nb-4d and V-3d orbitals, respectively. This orbital hybridization governs the fundamental electronic transitions and provides the microscopic basis for the observed optical response. Hybrid-functional (HSE06) calculations were employed to assess the sensitivity of the electronic structure to exchange–correlation effects. The results reveal pronounced band-edge renormalization near the Fermi level, particularly for transition-metal d states, underscoring the strong functional dependence of narrow-gap chalcogenide perovskites. These hybrid-functional results are therefore interpreted as a qualitative comparison rather than as a definitive reclassification of the electronic nature of the materials.

Optical properties, including dielectric functions, absorption coefficients, reflectivity, and optical conductivity, were consistently derived from the PBE-GGA electronic structures. Both compounds exhibit strong absorption in the visible and near-infrared regions, which can be directly correlated with the S-3p to transition-metal d interband transitions identified in the electronic structure analysis. A model-based thickness-dependent efficiency assessment was further used to compare relative optoelectronic trends; however, this analysis is not intended as a predictive device-performance evaluation.

In conclusion, this study provides a coherent and internally consistent description of the electronic and optical behavior of CsNbS_3_ and CsVS_3_, clarifying previously reported inconsistencies arising from methodological differences. The results highlight the sensitivity of transition-metal chalcogenide perovskites to exchange–correlation treatment and emphasize the importance of careful functional selection and interpretation. Future work incorporating phonon calculations, finite-temperature simulations, and many-body approaches would be required to establish definitive stability and device-level performance characteristics.

## Data Availability

The data supporting this study were generated using the Centre for High-Performance Computing (CHPC, South Africa) infrastructure. The data will be made available on request.
